# Less Common Variants of Posterior Semicircular Canal Benign Paroxysmal Positional Vertigo: Diagnostic and Therapeutic Considerations

**DOI:** 10.3390/jcm15010282

**Published:** 2025-12-30

**Authors:** Giuseppe Santopietro, Enrico Armato, Luigi Califano

**Affiliations:** 1 Dott. Giuseppe Santopietro Medical Practice, Audiology and Phoniatrics, 82100 Benevento, Italy; giuseppe.santopietro312@gmail.com; 2 Department of Neurosciences, University of Padova, 35100 Padova, Italy; armato.otovest@gmail.com; 3 Research Unit DevAH—Development, Adaptation and Handicap, Faculty of Medicine, University of Loraine, 54500 Vandoeuvre-lès-Nancy, France; 4 Department of Audiology and Phoniatrics, San Pio Hospital, 82100 Benevento, Italy

**Keywords:** vertigo, paroxysmal, posterior semicircular canal, BPPV variants, apogeotropic, Scocco, Yetiser, downbeating nystagmus

## Abstract

**Background:** Posterior canal benign paroxysmal positional vertigo (PSC-BPPV) is the most frequently encountered vestibular disorder in otoneurological clinical practice. However, diagnosis may be challenging in the presence of less common variants, which are characterised by atypical nystagmus patterns and may complicate clinical assessment. **Methods:** This study analysed a cohort of 295 patients diagnosed with PSC-BPPV and treated between January and August 2025 at the Audiology and Phoniatrics Unit of San Pio Hospital, Benevento. Of these, 25 patients presented with less common PSC-BPPV variants, namely the apogeotropic variant, the Scocco variant, and the Yetiser variant. Clinical features, therapeutic manoeuvres performed, time to symptom resolution, and associated comorbidities were evaluated. All patients were treated exclusively using the Epley manoeuvre. This observational study describes our experience in the diagnosis and management of these rare PSC-BPPV variants, with the aim of expanding the currently available evidence. **Results:** The Scocco variant was the only less common form of PSC-BPPV that required a statistically significantly greater number of liberatory manoeuvres when compared with classic PSC-BPPV (*p* < 0.05). No statistically significant differences were observed for the remaining variants with regard to treatment response or time to symptom resolution. **Conclusions:** Less common variants of PSC-BPPV represent both diagnostic and therapeutic challenges due to their atypical nystagmus patterns. Our findings indicate that the Scocco variant may be more resistant to standard liberatory treatment. In addition, this study reports auxiliary diagnostic and clinical tools which, in our experience, facilitated patient management.

## 1. Introduction

Benign paroxysmal positional vertigo (BPPV) represents the most prevalent vestibular disorder encountered in clinical practice, accounting for approximately 20–30% of all diagnoses [1,2]. In 1969, Schuknecht first described cupulolithiasis as the pathophysiological basis of BPPV [3]. This was followed by the description of canalolithiasis by Hall et al. and, shortly thereafter, by Epley in 1979 and 1980, respectively [4,5]. BPPV is frequently associated with psychiatric symptoms, which may adversely affect patients’ quality of life, and its diagnosis and management are of paramount importance for specialists in otoneurology [6].

The posterior semicircular canal (PSC) is the canal most frequently involved in canalolithiasis, representing 60/70% of all BPPV cases [7,8,9]. The PSC is probably the most frequently involved as it represents the most gravity-dependent portion of the vestibular endorgan [10]. In the typical geotropic form of PSC-BPPV, otoconia migrate into the ampullary arm of the canal, generating an ampullofugal endolymphatic flow that excites the cupula and induces a rotatory nystagmus with an upbeating vertical component. Diagnosis is established through positional testing, most commonly the Dix–Hallpike and Semont positioning tests [11,12].

In 2018, Choi et al. [13] introduced the Upright Protocol as a diagnostic approach designed to identify the affected canal and side with minimal vestibular stimulation. For the geotropic variant of PSC-BPPV, nystagmus is assessed during upright head flexion (Bowing) at 45° and extension (Leaning) at 45°. Leaning elicits a nystagmus pattern concordant with that observed during the Dix–Hallpike or Semont tests, whereas Bowing induces a reversal of the nystagmus direction. For example, an upbeating nystagmus with a clockwise torsional component in the Leaning position would be expected to reverse to a downbeating, counterclockwise nystagmus during Bowing, supporting the diagnosis of left-sided geotropic PSC-BPPV.

Management of BPPV consists of canalith-repositioning manoeuvres aimed at guiding otoconial debris through the effect of gravity, along the semicircular canal toward the utricular exit, which for the vertical canals corresponds to the common crus. The manoeuvres most commonly employed for PSC-BPPV are the Epley manoeuvre, described in 1990 [14], and the Semont manoeuvre, introduced in 1988 [10]. The Quick Liberatory Rotation manoeuvre, described by Califano et al. in 2003, has also proven highly effective in treating PSC-BPPV [15].

In recent years, several variants of posterior semicircular canal BPPV have been described. These variants arise from the specific positioning of otoconial debris within distinct segments of the PSC and, in some cases, from anatomical features intrinsic to the canal itself.

### 1.1. Apogeotropic Variant

In 1995, Agus et al. [16] first characterised an atypical nystagmus exhibiting a downbeating vertical component in patients with posterior semicircular canal benign paroxysmal positional vertigo (PSC-BPPV). To account for this clinical presentation, Agus proposed a model in which otoconia are situated within the non-ampullary arm of the affected canal. This anatomical placement generates an inhibitory ampullipetal endolymphatic current, thereby explaining the inverted characteristics of the diagnostic nystagmus.

Expanding upon this hypothesis, Vannucchi et al. (2012) [17] introduced the term ‘apogeotropic variant’ of PSC-BPPV, corroborating Agus’s assertion regarding the presence of debris in the non-ampullary arm.

Subsequently, in 2021, Califano et al. [18] postulated the existence of two distinct subgroups of apogeotropic PSC-BPPV. In this variant, otoconia may be located either in the non-ampullary arm or within the periampullary region. In both instances, diagnostic positioning induces an inhibitory ampullipetal current that elicits a downbeating torsional nystagmus. Notably, when debris is located in the periampullary region, the nystagmus may undergo a sudden conversion into a typical upbeating torsional pattern during head hyperextension in the Dix–Hallpike test; this phenomenon is attributed to the rapid migration of otoconia into the ampullary arm towards the most declivous part of the canal.

Furthermore, in 2014, Califano et al. [19] proposed a formal classification for apogeotropic PSC-BPPV, categorising the condition into possible, probable, and definite forms:

- Definite: The diagnosis is confirmed if conversion into typical geotropic PSC-BPPV is observed during positional testing.
- Probable: The diagnosis is likely if the disorder resolves following PSC liberatory manoeuvres, even in the absence of nystagmus conversion.
- Possible: The diagnosis is considered if resolution requires more than five liberatory manoeuvres, if the patient is lost to follow-up prior to resolution, or if magnetic resonance imaging (MRI) fails to identify central lesions that could otherwise explain the symptoms.

In 2015, Vannucchi et al. [20] developed two different physical therapy techniques for apogeotropic PSC-BPPV: the Demi-Semont manoeuvre and forced liberatory positioning.

**Figure 1:**
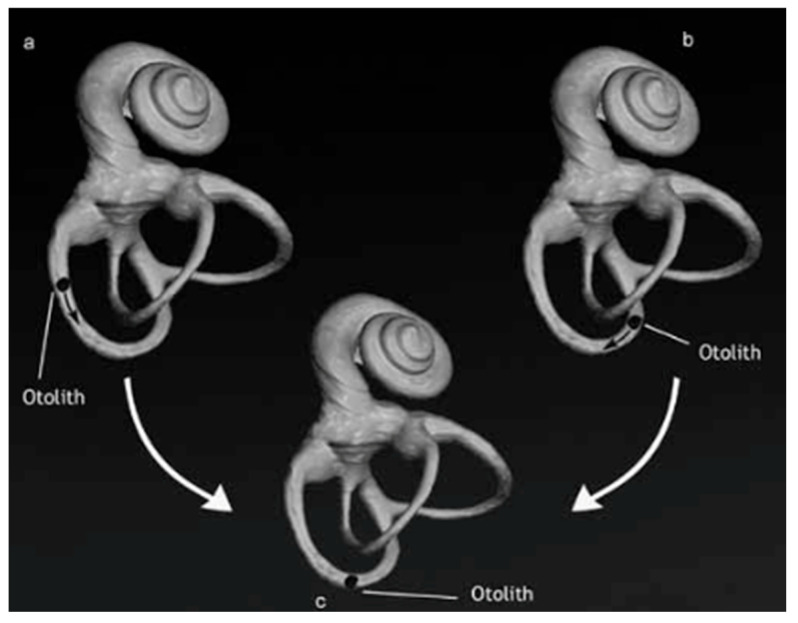
Left typical (**a**) and apogeotropic (**b**) posterior canal BPPV: the final position of otoliths in the Dix–Hallpike positioning test is the same, in the sloping part of the canal (**c**) [19].

In their paper, Califano et al. [19] stated that during the Dix–Hallpike diagnostic manoeuvre, otoconia assume an identical spatial configuration in both the geotropic and apogeotropic variants of PSC-BPPV (Figure 1). Consequently, once the Dix–Hallpike test has been performed on the affected side, the manoeuvres typically employed for standard PSC canalolithiasis—such as the Epley or Semont manoeuvres—are also applicable to the apogeotropic variant.

### 1.2. Short-Arm Canalolithiasis

In 2001, Oas provided the inaugural description of short-arm canalolithiasis affecting the posterior semicircular canal [21]. In this specific variant, otoconia sequester within the short arm of the canal on the utricular side of the cupula. When the patient is placed in the Dix–Hallpike position with the head hyperextended, the clinical presentation may include a subtle, persistent downbeating nystagmus with ipsidirectional torsion at the conclusion of the paroxysmal phase; this stands in contrast to the transient, inverted torsional nystagmus characteristic of long-arm canalolithiasis. Furthermore, during the execution of liberatory manoeuvres, a reversal of nystagmus may be elicited—a phenomenon not typically observed in the classic form of the condition.

### 1.3. Subjective BPPV and Type 2 BPPV

In certain instances, small otoconial particles fail to enter the posterior semicircular canal during the Dix–Hallpike manoeuvre. Instead, they sequester within the most dependent region of the vestibule, remaining in proximity to the utricle—a phenomenon potentially attributable to the elevated calcium ion (Ca^2+^) concentration in the peri-ampullary zone. This condition results in minimal, chronic ampullary stimulation, which is insufficient to elicit observable nystagmus during diagnostic testing.

However, upon returning to an upright seated position following the manoeuvre, patients may experience profound dizziness and a distinct sensation of retropulsion without nystagmus. It is therefore postulated that the otoconia are situated within or near the ampulla on its utricular aspect. During the transition from a seated to a Dix–Hallpike position, debris may be displaced from the ampulla, only to re-enter it when the patient returns to the sitting position. Clinical evidence suggests that repeated sit-up movements may prove effective in remediating this specific form [22,23].

This condition is formally designated as subjective BPPV. Many authorities posit that the so-called Type 2 BPPV, which manifests with comparable clinical features, represents the same pathological entity or a principal subtype. This variant is most frequently observed in patients assessed outside the acute phase or those exhibiting incomplete resolution of BPPV, and it is typically characterised by persistent postural symptoms.

### 1.4. Scocco Variant (Sitting-Up Nystagmus and Vertigo)

In 2019, Scocco described a new variant of posterior semicircular canal benign paroxysmal positional vertigo (PSC-BPPV), called “sitting-up nystagmus and vertigo variant” [24]. In individuals with this condition, no nystagmus or a small nystagmus is elicited during the Dix–Hallpike test; however, upon returning to the seated position, patients experience intense vertigo accompanied by a characteristic nystagmus pattern indicative of posterior semicircular canal involvement on the tested side.

According to Scocco’s hypothesis, otoconia may accumulate within the peri-ampullary region. Specific anatomical features of the canal—such as inherent narrowing (stenosis) or the large size of the otoconial mass—may impede the ampullofugal flow necessary to generate nystagmus during the Dix–Hallpike test. When the patient resumes a seated posture, gravity induces ampullofugal movement of the debris, thereby producing an excitatory cupular deflection. This deflection may persist due to a pseudo-continuous current generated by debris obstructing the stenotic segment of the canal. Consequently, the nystagmus, while characteristic of PSC-BPPV, deviates from the classic paroxysmal pattern and is elicited solely, or predominantly, upon the return to a sitting position.

In some clinical presentations, Scocco also observed a downbeating nystagmus during the Dix–Hallpike test. This finding may reflect debris movement towards the ampulla, which is positioned inferiorly relative to the peri-ampullary region in this specific posture. This observation lends support to Califano’s hypothesis regarding a peri-ampullary form of apogeotropic posterior semicircular canal BPPV [19].

The management of this variant involves standard liberatory manoeuvres indicated for PSC-BPPV. The application of mastoid vibration may prove beneficial in disaggregating large otoconial debris or facilitating their passage through canal stenosis. In clinical practice, the Scocco variant frequently represents the most challenging form to manage, often necessitating the implementation of home-based Brandt–Daroff exercises or the Bascule manoeuvre [25,26]. The primary differential diagnosis to consider is with postural hypotension and vertigo.

### 1.5. Heavy and Light Cupula

Heavy and light cupula conditions manifest when the density of the cupula deviates from that of the surrounding endolymph, rendering it relatively more or less buoyant. Under normal physiological conditions, the cupula and endolymph are isodense, ensuring that cupular deflection occurs exclusively in response to angular acceleration. However, when this buoyancy equilibrium is disrupted, the cupula becomes pathologically sensitive to linear acceleration and gravitational forces—stimuli typically mediated by the utricular and saccular maculae. The resulting nystagmus is characterised by its prolonged duration and minimal latency, often exhibiting direction-changing properties. Furthermore, a ‘neutral point’ may be identified, at which the cupula aligns vertically with the gravitational vector, resulting in an absence of deflection and a transient cessation of nystagmus [27,28].

The underlying pathophysiology of these conditions remains incompletely elucidated. It has been hypothesised that a light cupula may result from an increase in endolymph density—potentially due to suspended debris—or the introduction of low-density substances, as observed in alcohol-induced positional nystagmus [29,30]. Conversely, a heavy cupula may arise from the adhesion of otoconia to the cupula (cupulolithiasis) or alterations in endolymph composition. Nystagmus elicited during the Dix–Hallpike manoeuvre frequently resembles that of classic posterior semicircular canal benign paroxysmal positional vertigo (BPPV), yet it is distinguished by a notably longer duration and an absence of the characteristic latency [27].

Liberatory manoeuvres are generally ineffective when the condition is secondary to alterations in endolymph density. However, such interventions may prove beneficial—particularly when supplemented by mastoid vibration—if the underlying pathophysiology involves cupulolithiasis [19].

### 1.6. Yetiser Variant

In 2015, Yetiser described a novel variant of posterior semicircular canal benign paroxysmal positional vertigo (PSC-BPPV) [31]. The patient presented with a clinical history characteristic of the disorder; however, during the left Dix–Hallpike manoeuvre, a vertical–torsional nystagmus was observed with an upbeating vertical component and a counter-clockwise torsional component—features typically associated with right-sided geotropic PSC-BPPV. Furthermore, the nystagmus exhibited a secondary phase suggestive of left PSC involvement, indicated by clockwise torsion. Yetiser postulated that the otoconia were situated within the non-ampullary arm near the common crus, thereby generating two distinct endolymphatic currents:

1. An initial ampullipetal inhibitory current, producing an upbeating torsional nystagmus contralateral to the stimulated side.
2. A subsequent ampullofugal excitatory current, generating the characteristic upbeating torsional nystagmus ipsilateral to the side being tested.

This hypothesis was subsequently contested by Califano et al. [19]. Although a counter-clockwise torsional component during a left Dix–Hallpike manoeuvre could be attributed to an ampullipetal current, such a mechanism would theoretically necessitate a downbeating vertical component. The presence of an upbeating vertical component is, therefore, inconsistent with Ewald’s third law [32]. According to Ewald’s third law, within the posterior semicircular canal, an ampullopetal endolymphatic flow produces an inhibitory effect, giving rise to a downbeating nystagmus with an apogeotropic torsional component. In their paper, Califano et al. documented cases in which serial positional testing and liberatory manoeuvres led to the emergence of typical PSC-BPPV on the contralateral side, concomitant with the resolution of the atypical nystagmus. They proposed that, owing to an unusual anatomical orientation of the posterior semicircular canal, canal excitation may occur even when the patient is positioned on the contralateral side. Liberatory manoeuvres conventionally employed for typical PSC-BPPV have proven effective in the management of this variant [18,19].

## 2. Materials and Methods

### 2.1. Study Sample

From January 2025 to August 2025, at the Audiology and Phoniatrics Unit of the Hospital “San Pio” in Benevento 367 cases of acute benign paroxysmal positional vertigo (BPPV) were diagnosed and treated in accordance with current guidelines [33]. Of these, 80.38% (*n* = 295) involved posterior semicircular canal BPPV, consistent with data reported in the literature.

Twenty-five patients, representing 8.48% of the 295 posterior semicircular canal BPPV cases, presented with less common variants. The identified variants included apogeotropic PSC-BPPV, the Scocco variant, and the Yetiser variant. Cases meeting criteria for cupulolithiasis—using the additional criterion of enhanced nystagmus in the half Dix–Hallpike position—were included in the group of classical geotropic BPPV.

The sample comprised 13 males and 12 females (M:F ratio = 1:1), with a mean age of 63.24 ± 13.35 years (range: 30–82 years). Exclusion criteria included concomitant Ménière’s disease, a history of unilateral acute vestibulopathy, central vestibular disorders and the presence of neurodegenerative disorders such as amyotrophic lateral sclerosis or multiple sclerosis. Conversely, patients with post-traumatic BPPV were included.

For the comparative statistical analysis of the number of liberatory manoeuvres required to achieve effective resolution of the disorder, a control group of 25 patients diagnosed with typical PSC-BPPV was selected. Although 295 patients with posterior canal BPPV were evaluated, only a subset of 25 patients with typical PSC-BPPV was selected as the control group in order to ensure clinical and methodological homogeneity with the study groups affected by uncommon variants. The control group comprised 16 females and 9 males (M:F ratio = 1:2). The mean age of the control group was 62.56 ± 11.20 years (range: 36–82 years).

### 2.2. Diagnostic and Therapeutic Assessment

A meticulous medical history was obtained prior to clinical evaluation. All patients underwent a comprehensive vestibular assessment via bedside examination using binocular infrared video-oculoscopy (VOS) with integrated recording capabilities.

Spontaneous nystagmus, if present, was evaluated in the primary gaze position as well as in lateral, upward and downward gaze. Oculomotor function was assessed clinically, including saccadic movements, smooth pursuit, and vergence. The vestibulo-ocular reflex (VOR) was examined in darkness using VOS and subsequently under conditions of visual fixation to confirm its physiological suppression. Where benign paroxysmal positional vertigo (BPPV) was suspected based on clinical history, the Head Shaking Test (HST), clinical and video Head Impulse Test (cHIT and vHIT) were intentionally omitted during the initial assessment; the vigorous head movements required for a valid test may displace otoconial debris within the semicircular canals and potentially confound the original clinical presentation.

In cases of suspected BPPV, the ‘Upright Protocol’ was performed first—comprising the Head Pitch Test and Upright Head Roll Test—to evaluate nystagmus patterns associated with potential BPPV variants in the leaning and bowing positions. Diagnostic positioning manoeuvres were subsequently conducted, including the Pagnini–McClure (Head Yaw) Test and the Dix–Hallpike test.

For the apogeotropic variant of posterior semicircular canal (PSC) BPPV, we adopted the classification proposed by Califano et al. [18,19], as summarised in Table 1. To ensure therapeutic standardisation, the Epley manoeuvre was the exclusive intervention applied for all PSC-BPPV variants and for the control group. Mastoid vibration was applied in selected patients who showed resistance to liberatory manoeuvres, with the aim of fragmenting the otoliths and facilitating treatment of the BPPV. Brandt–Daroff or Bascule exercises were prescribed for patients who failed to respond after three therapeutic sessions. All patients were reviewed weekly until the complete resolution of nystagmus was achieved.

All patients with a history of recurrent BPPV underwent contrast-enhanced cranial MRI to exclude central pathologies.

### 2.3. Statistical Analysis

This study employs an observational–descriptive design. Continuous variables were compared between groups using the independent-samples Student’s *t*-test (for age) and the Mann–Whitney U test (for discrete or non-normally distributed variables, such as the number of liberatory manoeuvres). Categorical variables, such as sex distribution, were compared using Fisher’s exact test. All tests were two-tailed, and a *p*-value < 0.05 with a 95% confidence interval was considered statistically significant. For groups with very small sample sizes (*n* ≤ 3), results are presented descriptively, acknowledging the limited statistical power.

All statistical calculations were performed using Jamovi statistical software (Version 2.6.45.0; The Jamovi Project, Sydney, Australia).

### 2.4. Ethical Considerations

Approval from an Ethical committee was not required for this study, as it involved a retrospective review of anonymised clinical records. All procedures were conducted in accordance with the ethical standards of the institutional and national research committees, and strictly adhered to the principles of the Declaration of Helsinki.

## 3. Results

**Figure 2:**
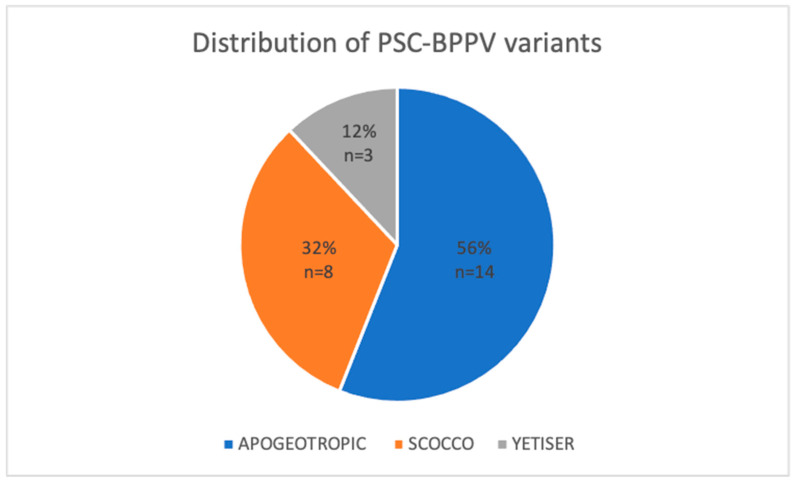
Distribution of less common variants of Posterior Semicircular Canal Benign Paroxysmal Positional Vertigo (PSC-BPPV).

Among the 295 cases of acute posterior semicircular canal benign paroxysmal positional vertigo (PSC-BPPV) analysed, 25 patients (8.48%) presented with less common variants. The distribution of these atypical presentations was as follows: 14 cases (56%) were classified as the apogeotropic form, 8 cases (32%) as the Scocco variant, and 3 cases (12%) as the Yetiser variant. Due to diagnostic uncertainty, three possible cases of light cupula and two cases of subjective BPPV were excluded. Figure 2 illustrates the proportional distribution of the study sample according to the specific diagnosed variant.

### 3.1. Apogeotropic PSC-BPPV

The mean age of the patient cohort was 64.08 ± 20.8 years (range: 30–79 years). Only one patient reported a history of head trauma within the five months preceding the onset of symptoms. Previous episodes of acute posterior or lateral canal BPPV were documented in 35.72% of cases (*n* = 5). One patient had a history of PSC-BPPV variants, specifically the Scocco variant and apogeotropic PSC-BPPV; in the remaining eight cases, the current episode represented the initial presentation of the disorder.

**Figure 3:**
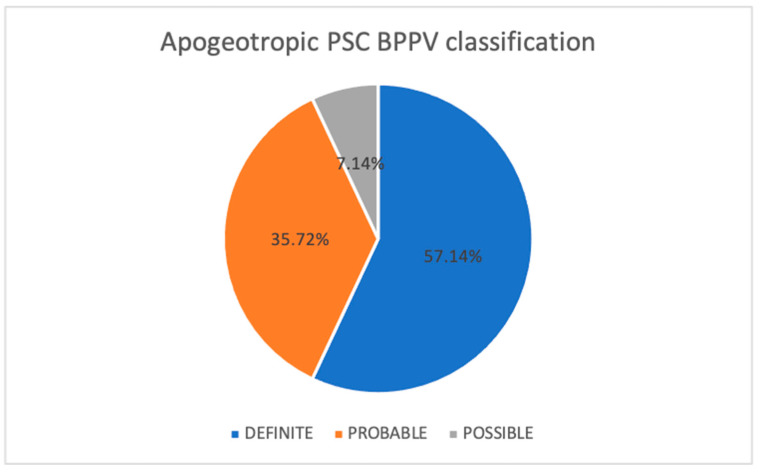
Classification of apogeotropic PSC-BPPV in our series.

Cranial magnetic resonance was performed in patients with a history of recurrent BPPV, with no significant findings reported. Cases of apogeotropic PSC-BPPV were classified according to the criteria established by Califano et al. [18,19] (Table 1). Within our sample, geotropisation of the nystagmus—confirming a diagnosis of ‘definite’ apogeotropic PSC-BPPV—was observed in 57.14% of cases (*n* = 8). In five cases (35.72%), the diagnosis was considered ‘probable’, as the disorder resolved following liberatory manoeuvres despite the absence of geotropisation. One case (7.14%) was classified as ‘possible’, as the patient was lost to follow-up prior to clinical resolution. Figure 3 illustrates the distribution of cases according to these classification categories.

Diagnosis was established in all 14 patients via the Dix–Hallpike test and subsequent analysis of the elicited nystagmus. The Upright Protocol facilitated a diagnosis in only eight of the 14 apogeotropic PSC-BPPV cases (57.14%) and proved inconclusive for the remaining patients.

Among the eight patients who underwent geotropisation, five achieved resolution after a single liberatory manoeuvre, presenting with no residual signs of BPPV at the seven-day follow-up. Within this group, three patients exhibited liberatory nystagmus during the Epley manoeuvre, whereas in two cases, the disorder resolved despite the absence of liberatory nystagmus, as confirmed by negative Dix–Hallpike and Pagnini–McClure test at follow-up. The remaining three patients required two Epley manoeuvres for resolution.

One patient did not receive any liberatory manoeuvre during the initial consultation due to severe autonomic symptoms. At the second assessment, geotropisation occurred following a demi-Semont manoeuvre, which was immediately succeeded by an Epley manoeuvre. A third Epley manoeuvre was performed at the subsequent visit for residual classic PSC-BPPV, after which complete resolution was achieved.

Another notable case involved a patient presenting with simultaneous apogeotropic PSC-BPPV and apogeotropic lateral canal BPPV (LC-BPPV) on the same side. At the initial evaluation, priority was given to the management of LC-BPPV, which was associated with a greater impairment of the patient’s quality of life. Following mastoid vibration, a modified Gufoni manoeuvre was performed on the affected side; owing to a lack of geotropisation, this was followed by forced liberatory positioning according to Vannucchi. At the one-week follow-up, the LC-BPPV had transformed into a geotropic form, and geotropisation of the ipsilateral PSC-BPPV was also observed. The PSC-BPPV resolved after an Epley manoeuvre, and the LC-BPPV was successfully treated with a Gufoni manoeuvre during the same session.

In the ‘probable’ group, only one patient required more than one liberatory manoeuvre (three in total) to achieve resolution. Excluding the patient lost to follow-up, the mean number of liberatory manoeuvres required to treat apogeotropic PSC-BPPV was 1.39 ± 0.74 (range: 1–3).

### 3.2. Scocco Variant

The mean age of the patient cohort was 67.88 ± 10.2 years, with an equal sex distribution. One patient had a medical history of migraine without vestibular symptoms. Half of the cohort (*n* = 4) had a history of recurrent PSC-BPPV on the same side, consistently presenting in the geotropic form; these individuals had previously undergone contrast-enhanced cranial MRI and serum vitamin D assessment, with no abnormal findings. Two patients had experienced only a single prior episode of typical PSC-BPPV, one of which occurred a decade earlier. In the remaining two cases, the Scocco variant represented the initial presentation of BPPV.

In four of the eight cases, the Dix–Hallpike manoeuvre elicited a downbeating nystagmus, followed by a typical upbeating nystagmus with either a clockwise or counter-clockwise torsional component upon returning to the seated position, depending on the side assessed. In the other four cases, nystagmus was observed exclusively upon returning to the seated position. One patient with a right-sided Scocco variant exhibited an upbeating, counter-clockwise nystagmus during the Half Dix–Hallpike manoeuvre, which was consistent with the findings elicited upon sitting up.

Among the four patients whose condition resolved with the Epley manoeuvre, the mean number of manoeuvres required was 2.5 ± 1.06 (range: 1–4). Four patients were prescribed home-based Brandt–Daroff exercises for a duration of 15 days; three achieved complete resolution at the 15-day follow-up, while the remaining patient required two additional Epley manoeuvres to achieve resolution. One case was identified as a secondary Scocco variant, diagnosed at follow-up after the treatment of classic PSC-BPPV; this was successfully managed with three Epley manoeuvres and Brandt–Daroff exercises.

Another notable case involved a 60-year-old woman initially diagnosed with a right-sided Scocco variant. At the subsequent consultation, she developed a contralateral Scocco variant. Treatment was first directed towards the side originally affected, requiring two manoeuvres, followed by the contralateral side, which required three manoeuvres in addition to Brandt–Daroff exercises to achieve complete clinical resolution.

### 3.3. Yetiser Variant

The Yetiser variant was diagnosed in three cases (12%), with a mean age of 50.33 ± 23.6 years. One patient developed BPPV following head trauma, while the remaining two cases were idiopathic. The mean number of liberatory manoeuvres required was 1.33 ± 0.58 (range: 1–2).

In all three cases, serial diagnostic positioning (repeated two to five times) induced a conversion into typical PSC-BPPV, with nystagmus elicited exclusively on the affected side. Resolution was achieved after a single Epley manoeuvre in two cases, while the third individual required two sessions conducted one week apart. The Yetiser variant represented the initial presentation of BPPV in only one patient; the other two individuals had a history of previous typical PSC-BPPV episodes. 

One patient subsequently experienced two further BPPV episodes—a geotropic lateral canal BPPV (LC-BPPV) and a typical PSC-BPPV—both of which were ipsilateral and successfully managed. Vitamin D assessment and contrast-enhanced cranial MRI were performed to exclude deficiency or central aetiologies, yielding unremarkable results.

### 3.4. Typical PSC-BPPV

In the control group, the diagnosis of typical PSC-BPPV was established using the Upright protocol and the Dix–Hallpike positioning manoeuvre. The mean number of Epley manoeuvres required to achieve resolution of the condition was 1.48 ± 0.58 (range: 1–2).

### 3.5. Statistical Comparison

A comparison of the mean number of liberatory manoeuvres required for the different PSC-BPPV variants versus the control group (typical PSC-BPPV) revealed no statistically significant difference for the apogeotropic and Yetiser variants (*p* = 0.31 and *p* = 0.73, respectively). In contrast, for the Scocco variant, the Mann–Whitney U-test yielded a *p*-value of 0.0086 in the comparison of the number of liberatory manoeuvres required versus those required in a cohort of 8 patients with geotropic PSC-BPPV. This finding confirms—consistent with clinical observational impressions—that this specific variant generally necessitates a higher number of manoeuvres and, in certain cases, adjunctive home exercises for effective therapeutic management. Statistical analysis revealed no significant differences between the study and control groups in terms of mean age and sex distribution (*p* = 0.85 and *p* = 0.26, respectively). Table 2 presents the data analysed in the statistical study for the case and control groups.

## 4. Discussion

Our findings indicate that uncommon forms of PSC-BPPV may be more prevalent than commonly recognised in clinical practice. In our cohort, these variants accounted for 8.48% of the 295 patients diagnosed with PSC-BPPV. Overall, the variants identified and treated in this study demonstrated a favourable response to liberatory manoeuvres, with clinical resolution rates largely comparable to those observed in typical PSC-BPPV in the apogeotropic and Yetiser variants. In contrast, the Scocco variant was associated with the need for a significantly higher number of liberatory manoeuvres to achieve symptom resolution, suggesting a potentially greater therapeutic complexity for this specific subtype.

In 1995, Epley proposed that any nystagmus not fulfilling the classical Dix–Hallpike criteria should be considered atypical [34]. In typical PSC canalolithiasis debris movement away from the cupula generates a geotropic torsional upbeat nystagmus with a short latency and duration, which occurs when the affected ear is positioned downward during the Dix–Hallpike manoeuvre. Upon returning to the upright position from the head-hanging posture, the nystagmus typically reverses direction, producing a downbeat nystagmus as the debris change the direction of their movement. In cupulolithiasis, nystagmus is typically more sustained and often persists throughout the entire Dix–Hallpike manoeuvre [35,36,37,38]. Such atypical presentations may arise from abnormal sequestration of otoconial debris or, in certain cases, from anatomical variations in the semicircular canals. These mechanisms can generate nystagmus patterns that diverge from canonical descriptions while remaining compatible with Ewald’s laws [32]. Among the recognised variants of posterior semicircular canal (PSC) benign paroxysmal positional vertigo (BPPV), the apogeotropic form appears to be the most prevalent. In our cohort, it accounted for 4.74% of all PSC-BPPV cases and 56% of the less common variants.

The principal challenge in apogeotropic PSC-BPPV lies in its clinical identification. Careful and systematic history-taking is essential prior to positional testing, as BPPV is characterised by distinctive clinical features that often allow preliminary diagnostic orientation. This approach enables a more focused and less distressing examination for the patient. In PSC-BPPV, patients typically experience brief, intense vertigo episodes, often triggered by rising from bed or by upward and downward head movements. These episodes are frequently accompanied by nausea and vomiting, and may lead to transient postural instability. Recurrent vertigo upon repeated head movements further underscores the positional nature of the condition and the susceptibility of the affected canal. In less common variants, however, the clinical history may deviate substantially from typical presentations. For example, in the Scocco variant, symptoms occur predominantly upon sitting up, whereas in the more common apogeotropic form, lateralisation is frequently unclear and dizziness may predominate over true rotational vertigo. Awareness of these atypical forms is therefore crucial for timely and accurate diagnosis.

In our series, the classical Dix–Hallpike manoeuvre demonstrated high sensitivity in eliciting nystagmus in atypical PSC-BPPV. By contrast, the Upright Protocol was less sensitive for PSC involvement than for lateral canal BPPV. Whereas lateral canal BPPV typically produces nystagmus in nearly all patients during upright positioning, this response was not consistently observed in PSC-BPPV. Martellucci et al. reported that PSC-BPPV could be correctly diagnosed in 75.2% of cases using the Head Pitch Test alone, with diagnostic accuracy increasing to 87.5% when stimulation in the RALP and LARP planes was added [39]. In our cohort, however, the diagnostic yield of the Upright Protocol in less common variants was lower, being positive in only 8/25 patients (32%). Moreover, the induced positional nystagmus—characterised by a downbeating vertical component with torsion opposite to that observed in typical PSC-BPPV—was not easily distinguishable from that produced by stimulation of the contralateral anterior canal.

Differentiation between apogeotropic PSC-BPPV and contralateral anterior canal BPPV remains a significant diagnostic challenge. In both anterior canal BPPV (AC-BPPV) and apogeotropic PSC-BPPV, nystagmus can be elicited bilaterally during the Dix–Hallpike manoeuvre and in the head-hanging position. In apogeotropic PSC-BPPV, the torsional component is typically more pronounced, whereas in AC-BPPV it may be minimal or absent, with a predominant vertical downbeat component. Importantly, performing the head-hanging manoeuvre may not only facilitate the resolution of AC-BPPV but can also convert apogeotropic PSC-BPPV to its typical geotropic form [40]. Califano et al. [19] suggested that a diagnosis of definite apogeotropic PSC involvement can only be established when geotropisation occurs, manifested by conversion to the typical PSC-BPPV pattern [18,19]. Only under these circumstances—analogously to anterior canalithiasis—can a peripheral origin of downbeating nystagmus be confidently established. Castellucci et al. subsequently proposed the video Head Impulse Test (vHIT) as a valuable adjunct in this differential diagnosis [41]. In their cohort of 59 patients with downbeating positional nystagmus, reduced vestibulo-ocular reflex (VOR) gain in the affected canal was identified in 72.9% of cases, rising to 88.6% among those with persistent or spontaneous downbeating nystagmus. These findings may reflect partial or complete canalith jam in atypical variants, whereas vHIT results in typical PSC-BPPV are generally within normal limits.

In our series, patients who underwent geotropisation during the initial evaluation required fewer liberatory manoeuvres than those who did not (mean 1.33 ± 0.58). This observation suggests that moving otoconia towards the ampullary arm is not only critical for diagnostic confirmation but also facilitates more efficient treatment. In several cases, geotropisation occurred during diagnostic positioning, sometimes following mastoid vibration, Head Shaking, or transition from the Half Dix–Hallpike to the full Dix–Hallpike position. These phenomena are likely attributable to the sudden downward displacement of debris initially located in a peri-ampullary position (Type 2 apogeotropic PSC-BPPV). When geotropisation occurred only after liberatory manoeuvres, the debris was probably located in the non-ampullary arm, as originally proposed by Agus [16]. The mechanisms leading to geotropisation in our cohort are summarised in Table 3, with mastoid vibration appearing particularly effective in disrupting otoconial aggregates, especially in cases of canalith jam.

**Figure 4:**
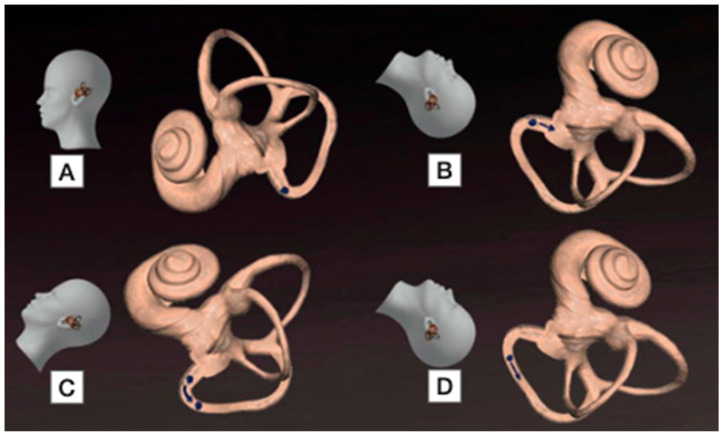
The Half Dix–Hallpike position to facilitate geotropisation moving otoconial debris. (**A**). During the Dix-Hallpike test they move ampullopetally (**B**). A quick and limited bowing of the head (**C**) followed by head hyperextension cause otoconia to move ampullophugally as in typical posterior canal BPPV (**D**) [18].

The Half Dix–Hallpike position also proved effective in promoting geotropisation. In peri-ampullary forms, the Dix–Hallpike position with head extension directs debris towards the ampulla, producing an ampullipetal inhibitory stimulus. Maintaining this position—or transitioning through the Half Dix–Hallpike—may subsequently allow debris to migrate towards the dependent segment of the canal, generating an ampullofugal excitatory stimulus and thereby enabling geotropisation. This proposed mechanism is illustrated in Figure 4. In cases of probable apogeotropic PSC-BPPV, Califano et al. hypothesised that immediate canal clearance may occur when debris is located on the utricular side of the ampulla [19]. In such instances, a brief downbeating or apogeotropic nystagmus may be observed immediately following a liberatory manoeuvre, as debris traverses the distal portion of the canal, thereby confirming its prior location in the non-ampullary arm. One patient in our cohort presented with recurrent PSC-BPPV manifesting exclusively as atypical variants, namely the Scocco and apogeotropic forms. Although a canal abnormality or atypical spatial orientation was suspected, both magnetic resonance imaging and temporal bone computed tomography were unremarkable. Nevertheless, such anatomical hypotheses cannot be entirely excluded, given the inherent limitations of morphological assessment of the semicircular canals.

The clinical features observed in our cases of the Scocco variant were consistent with those reported in the literature. These patients required longer treatment courses than other variants, with a mean of 2.5 ± 1.06 liberatory manoeuvres, and frequently required adjunctive Brandt–Daroff exercises. The difficulty in achieving canal clearance may result from impaired debris migration from the ampullary arm towards the common crus, possibly due to large otoconial aggregates or peri-ampullary stenosis. Early prescription of Brandt–Daroff exercises may therefore be advisable in this variant to promote debris fragmentation.

With respect to the Yetiser variant, the original pathophysiological explanation appears improbable, as it contradicts Ewald’s laws by postulating simultaneous excitatory and inhibitory responses. Califano et al. instead proposed that this variant may arise from an atypical spatial orientation of the PSC, allowing canal stimulation even during contralateral positioning [19]. In our cases, atypical nystagmus was observed on one side, followed by typical PSC-BPPV nystagmus on the opposite side, with subsequent disappearance of the atypical response. This observation supports the hypothesis of abnormal canal orientation and may also account for recurrent episodes in one of our patients.

Finally, it is essential to acknowledge the existence of central positional nystagmus (CPN), which is elicited by changes in head position relative to gravity and may closely mimic benign paroxysmal positional vertigo (BPPV). CPN can occur either paroxysmally or persistently, presenting as torsional downbeating nystagmus in the head-hanging position or apogeotropic nystagmus in Dix–Hallpike positions, with occasional overlap of both patterns. Clinically, CPN is often accompanied by additional central neurological signs, particularly cerebellar ocular motor deficits. It typically does not respond to liberatory manoeuvres [42]. Such presentations are most commonly associated with cerebellar lesions [43,44,45]. In our view, rigorous weekly follow-up is crucial to minimise the risk of misdiagnosis. Systematic monitoring of clinical progression and response to therapeutic manoeuvres is fundamental in distinguishing peripheral from central aetiologies. Diagnostic certainty regarding the peripheral origin of downbeating nystagmus can only be achieved when the nystagmus converts to a characteristic pattern of posterior (or lateral canal) BPPV, corresponding to the definitive grade as outlined in Califano’s classification [19].

Although cranial magnetic resonance imaging is indicated when a central origin is suspected, imaging may be unremarkable in conditions such as vestibular migraine, in which positional nystagmus has been reported in up to 90% of acute episodes [42,45]. Male et al. [46] have suggested transient ion channel dysfunction or altered neural excitability as potential pathophysiological mechanisms underlying these findings.

Consequently, meticulous history-taking, comprehensive clinical assessment, and strict follow-up—particularly in cases of atypical BPPV—remain essential for ensuring diagnostic accuracy.

## 5. Conclusions

A comprehensive understanding of the less common variants of posterior semicircular canal BPPV, alongside a solid grasp of Ewald’s laws, is essential for clinicians in accurately diagnosing atypical presentations of benign paroxysmal positional vertigo. In all these less common variants, the fundamental principles governing the generation and direction of nystagmus remain consistent. Although these atypical variants account for a minority of clinical presentations, they pose significant diagnostic and therapeutic challenges. Our study highlights that the apogeotropic form is the most prevalent of these variants, often requiring geotropisation, which can be facilitated by techniques such as mastoid vibration or the Half Dix–Hallpike position, to establish a definitive peripheral diagnosis. While the apogeotropic and Yetiser variants demonstrate a therapeutic response similar to that of classic posterior semicircular canal BPPV, the Scocco variant is notably more refractory, often necessitating a greater number of liberatory manoeuvres and adjunctive home-based exercises for resolution. A meticulous clinical approach, combining thorough history-taking with systematic positional testing and regular follow-up, is vital for the effective management of these variants. However, vertical downbeating nystagmus should always raise suspicion and prompt careful evaluation. Such vigilance not only ensures the successful resolution of atypical peripheral disorders but also acts as a critical safeguard against the misdiagnosis of central vestibular pathologies.

## Figures and Tables

**Table 1:** Classification proposed by Califano et al. for Apogeotropic PSC-BPPV [19]. APC BPPV: apogeotropic posterior benign paroxysmal positional vertigo; MRI: magnetic resonance imaging.

**(a) Definite APC BPPV**
Presence of torsional apogeotropic nystagmus, evoked through the Dix-Hallpike test and sometimes through the straight head hanging positioning Presence of a vertical down-beating component in the same positioning tests Canalar conversion in typical posterior canal BPPV during or immediately after (no more than two days) the therapeutic manoeuvre, or suddenly during the prolonged maintenance of one of the head hanging positions (straight head hanging; “deep” Dix-Hallpike position) or during the maintenance of leaning position in sitting position
**(b) Probable APC BPPV**
As reported in a, but with a direct resolution of disease without canalar conversion in typical posterior canal BPPV
**(c) Possible APC BPPV**
Persistence of symptoms after 5 cycles of therapeutic manoeuvres MRI does not show any structural alterations of the central nervous system as a presumed cause of the nystagmus or Patient lost to follow-up before the resolution of the disease

**Table 2:** Study and control group data. Colours indicate distribution of PSC-BPPV variants in the study sample: green—Apogeotropic variant; orange—Scocco variant; yellow—Yetiser variant.

Group A: Less Common Variants PSC-BPPV			Group B: Typical PSC-BPPV		
Sex	Age	n. Epley Manouvres	Sex	Age	n. Epley Manouvres
F	61	1	F	55	1
F	63	3	F	64	1
F	75	1	F	78	1
F	54	1	M	63	1
M	70	2	M	62	2
M	75	1	M	60	3
F	54	3	M	51	1
F	61	1	F	45	1
M	79	1	F	48	2
M	67	1	F	57	1
M	71	1	F	82	1
M	65	1	F	66	2
F	62	1	F	67	2
M	30	1	F	63	2
M	64	4	F	58	1
M	65	1	M	36	2
F	51	2	F	70	1
M	82	3	F	73	1
F	60	2	M	69	1
F	71	4	F	74	1
M	80	2	F	75	2
F	70	2	F	64	2
F	32	2	M	78	2
M	42	1	M	60	1
M	77	1	M	46	2
M:F = 1:1	Mean: 63.24 ± 13.35	Mean: 1.72 ± 0.96	M:F = 1:2	Mean: 62.56 ± 11.20	Mean: 1.48 ± 0.58

**Table 3:** Geotropisation in Definite Apogeotropic PSC-BPPV.

Definite Apogeotropic PSC BPPV	Geotropization	Position of Otoliths
1	Mastoid vibration + Half-Hallpike	Periampullary
2	Half-Hallpike	Periampullary
3	Home reclining	Non-ampullary arm
4	Supine Head shaking and head hyperextension	Periampullary
5	Short-Epley toward the pathological side in suspected ASC BPPV	Non-ampullary arm
6	Dix-Hallpike	Periampullary
7	Demi-Semont	Non-ampullary arm
8	Dix-Hallpike	Periampullary

## Data Availability

Data are available from the corresponding author upon reasonable request.
